# Cover crop mixture diversity, biomass productivity, weed suppression, and stability

**DOI:** 10.1371/journal.pone.0206195

**Published:** 2019-03-14

**Authors:** A. M. Florence, L. G. Higley, R. A. Drijber, C. A. Francis, J. L. Lindquist

**Affiliations:** 1 Department of Agronomy and Horticulture, University of Nebraska-Lincoln, Lincoln, Nebraska, United States of America; 2 School of Natural Resources, University of Nebraska-Lincoln, Lincoln, Nebraska, United States of America; USDA Agricultural Research Service, UNITED STATES

## Abstract

The diversity-productivity, diversity-invasibility, and diversity-stability hypotheses propose that increasing species diversity should lead, respectively, to increased average biomass productivity, invasion resistance, and stability. We tested these three hypotheses in the context of cover crop mixtures, evaluating the effects of increasing cover crop mixture diversity on aboveground biomass, weed suppression, and biomass stability. Twenty to forty cover crop treatments were replicated three or four times at eleven sites using eighteen species representing three cover crop species each from six pre-defined functional groups: cool-season grasses, cool-season legumes, cool-season brassicas, warm-season grasses, warm-season legumes, and warm-season broadleaves. Each species was seeded as a pure stand, and the most diverse treatment contained all eighteen species. Remaining treatments included treatments representing intermediate levels of cover crop species and functional richness and a no cover crop control. Cover crop seeding dates ranged from late July to late September with both cover crop and weed aboveground biomass being sampled prior to winterkill. Stability was assessed by evaluating the variability in cover crop biomass for each treatment across plots within each site. While increasing cover crop mixture diversity was associated with increased average aboveground biomass, we assert that this was the result of the average biomass of the pure stands being drawn down by low biomass species rather than due to niche complementarity or increased resource use efficiency. At no site did the highest biomass mixture produce more than the highest biomass pure stand. Furthermore, while increases in cover crop mixture diversity were correlated with increases in weed suppression and biomass stability, we argue that this was largely the result of diversity co-varying with aboveground biomass, and that differences in aboveground biomass rather than differences in diversity drove the differences observed in weed suppression and stability.

## Introduction

Increasing species diversity is thought to lead to increased average productivity, invasion resistance, and stability [1,2]. Respectively named the diversity-productivity, diversity-invasibility, and diversity-stability hypotheses, these hypotheses, while contested in the field of ecology [3–5], have often been treated in the field of agriculture as proven principle with regard to mixed cropping despite a lack of compelling empirical evidence in favor of these assertions [6–9]. The goal of this study is to test these hypotheses in the context of cover crop mixtures.

Cover crops are used to provide a variety of functions, many of which are positively related to cover crop productivity. These functions include weed suppression, soil nutrient retention, soil erosion control, and organic matter addition. While cover crops have been used for a long time for soil and crop benefits, the use of highly diverse cover crop mixtures is a relatively recent phenomenon. It has been suggested that by increasing cover crop mixture diversity, the various functions of cover crops will be enhanced and stabilized. Specifically, it has been proposed in both the popular press and the scientific literature that increasing cover crop mixture diversity should be associated with increased productivity, weed suppression, and biomass stability—claims that parallel the assertions made by the diversity-productivity, diversity-invasibility, and diversity-stability hypotheses (e.g. [10–14]). The popular press on cover crop mixtures suggests that a cover crop mixture can be more productive than a single cover crop species [10–11]. However, the majority of previous plant mixture studies in agriculture and ecology have found that while the average productivity of mixtures often exceeds the average productivity of the constituent species in pure stands, the most productive mixture is not necessarily more productive than the most productive single species [7,14–17].

While the diversity-productivity, diversity-invasibility, and diversity-stability hypotheses may appear to address three distinct topics, niche differentiation between species is used as the logical basis for all of them. Niche differentiation implies that different species have different resource needs and acquisition abilities. A single species is expected to leave resources unexploited that another species might be able to exploit—e.g., through its differential root or canopy architecture. Thus, the diversity-productivity hypothesis expects that a more diverse system should be more productive than a less diverse system due to increased resource use efficiency or niche complementarity [18]. Also, since a more diverse community is expected to fully use the finite resources in an environment than a less diverse community, the diversity-invasibility hypothesis predicts that a more diverse community will also be less susceptible to invasion by other species than a less diverse community, as more of the available resources have been pre-empted [5]. Furthermore, since different species have different resource requirements and physiological efficiencies, it follows that different species will thrive and fail under different conditions. As a result, the diversity-stability hypothesis predicts that the presence of many species insures that at least some species will thrive under variable environmental conditions, thereby stabilizing the performance of the species mixture [4].

Using cover crop mixtures as our model system, we ask with this study: Does increasing cover crop mixture diversity (1) increase cover crop biomass productivity, (2) increase weed suppression, and/or (3) increase biomass stability?

## Materials and methods

### Research sites

This study was conducted at eleven sites on farms across southeastern Nebraska. Cover crops were seeded at various times in a variety of crop rotations (Table 1). With the exception of sites 1 and 4, where the farm was irrigated, all other sites were rain-fed.

**Table 1:** Study locations, seeding dates, seeding conditions, and sampling dates. In cases where cover crops were seeded into a maturing crop, the growth stage of that crop is also provided in parentheses.

Site	Location*	Cover crop seeding date	Seeding conditions	Sampling date^†^
1	40°24'60"N 99° 2'60"W	7/19/2013	Wheat stubble	-
2	40°58'25"N 97°59'15"W	8/10/2013	Barley stubble	-
3	41°40'15"N 96°33'45"W	8/31/2013	Wheat stubble (disked)	10/31/2013
4	41°10'20"N 96°27'30"W	9/10/2013	Soybeans (R5)	11/9/2013
5	41°40'10"N 96°33'50"W	9/12/2013	Soybeans (R7)	11/7/2013
6	41°40'20"N 96°34'5"W	9/12/2013	Corn (R6)	-
7	40°58'10"N 97°59'50"W	9/14/2013	Soybeans (R6)	11/14/2013
8	41°19'45"N 96°16'55"W	9/19/2013	Corn stubble (disked)	11/8/2013
9	40°19'5"N 98°35'45"W	9/20/2013	Corn (R6)	-
10	41°40'20"N 96°33'40"W	7/20/2014	Wheat stubble (disked)	9/27/2014
11	40°51'5"N 96°28'10"W	7/23/2014	Wheat stubble	10/14-15/2014

*Studies on private land were conducted with the consent of the land-owner. This research did not involve endangered or protected species.

^**†**^Not all sites were sampled for plant biomass.

### Treatments

The study was started in 2013 with twenty treatments representing pure stands and mixtures of nine species—barley (*Hordeum vulgare* L.), oat (*Avena sativa* L.), wheat (*Triticum aestivum* L.), Austrian winter pea (*Pisum sativum* L. ssp. s*ativum* var. *arvense*), red clover (*Trifolium pratense* L.), yellow sweetclover (*Melilotus officinalis* (L.) Lam.), radish (*Raphanus sativus* L.), rapeseed (*Brassica napus* L. var. *napus*), and turnip (*Brassica rapa* L. var. *rapa*) (Table 2). The nine species were selected to represent three functional groups—cool-season grasses, legumes, and brassicas. The grasses used were spring varieties, which winterkilled with the legumes and brassicas.

**Table 2:** Summary of cover crop treatments for 2013.

No.	Functional group(s)	Treatment	No. of species	No. of groups
1	-	No cover	0	0
2	Cool-season grasses (CG)	Barley (BAR)	1	1
3		Oats (OAT)	1	1
4		Wheat (WHT)	1	1
5	Cool-season legumes (CL)	Austrian winter pea (PEA)	1	1
6		Red clover (RED)	1	1
7		Yellow sweetclover (YEL)	1	1
8	Cool-season brassicas (CB)	Radish (RAD)	1	1
9		Rapeseed (RAPE)	1	1
10		Turnip (TURN)	1	1
11	CG	BAR + OAT + WHT	3	1
12	CL	PEA + RED + YEL	3	1
13	CB	RAD + RAPE + TURN	3	1
14	CG + CL	BAR + OAT + WHT + PEA + RED + YEL	6	2
15	CG + CB	BAR + OAT + WHT + RAD + RAPE + TURN	6	2
16	CL + CB	PEA + RED + YEL + RAD + RAPE + TURN	6	2
17	CG + CL + CB	All 9 cool-season species	9	3
18	CG + CL + CB	BAR + PEA + RAD	3	3
19		OAT + RED + RAPE	3	3
20		WHT + YEL + TURN	3	3

Treatment 1 was a no cover control. Treatments 2–10 were pure stands. Treatments 11–13 were mixtures of all three cool-season grasses, legumes, and brassicas, respectively. These treatments served to evaluate the effect of increasing species diversity without increasing functional diversity.

Treatment 14 combined grasses with legumes, treatment 15 combined legumes with brassicas, and treatment 16 combined grasses with brassicas. These treatments served as a level of functional diversity intermediate between treatments 11–13 and treatment 17, which combined all nine species.

Treatments 18–20 were combinations of one grass, one legume, and one brassica. These treatments were designed so that each of the nine species was present in one of the three treatments. In designing the treatments, a point was made to make sure that each species was equally represented at each level of species and functional richness to address the issue of sampling bias—that is, the issue that as diversity increases, the likelihood of a certain species being included also increases [19–21]. Beyond that criterion, the specific combination of each grass, legume, and brassica was arbitrary.

In 2014, the study was expanded to include an additional 20 treatments (Table 3). Of these additional treatments, treatments 21–39 represented warm-season analogues of treatments 2–20. That is, warm-season grasses, legumes, and broadleaves were used instead of the cool-season grasses, legumes, and brassicas. The species used were proso millet (*Panicum miliaceum* L.), sorghum sudangrass (*Sorghum bicolor* (L.) Moench x *Sorghum bicolor* (L.) Moench var. Sudanese), teff (*Eragrostis tef* (Zuccagni) Trotter), chickpea (*Cicer arietinum* L.), cowpea (*Vigna unguiculata* (L.) Walp.), sunnhemp (*Crotalaria juncea* L.), buckwheat (*Fagopyrum esculentum* Moench), safflower (*Carthamus tinctorius* L.), and sunflower (*Helianthus annuus* L.). Treatment 40 was a combination of the original nine cool-season species and nine warm-season species. For a discussion of the traits associated with the cover crop species used in this study, refer to Clark [22].

**Table 3:** Summary of cover crop treatments added in 2014.

No.	Functional group(s)	Treatment	No. of species	No. of groups
21	Warm-season grasses (WG)	Proso millet (PROSO)	1	1
22		Sorghum sudangrass (SORG)	1	1
23		Teff (TEFF)	1	1
24	Warm-season legumes (WL)	Chickpea (CHICK)	1	1
25		Cowpea (COW)	1	1
26		Sunn hemp (SUNN)	1	1
27	Warm-season broadleaves (CB)	Buckwheat (BUCK)	1	1
28		Safflower (SAFF)	1	1
29		Sunflower (SUNF)	1	1
30	WG	PROSO + SORG + TEFF	3	1
31	WL	CHICK + COW + SUNN	3	1
32	WB	BUCK + SAFF + SUNF	3	1
33	WG + WL	PROSO + SORG + TEFF + CHICK + COW + SUNN	6	2
34	WG + WB	PROSO + SORG + TEFF + BUCK + SAFF + SUNF	6	2
35	WL + WB	CHICK + COW + SUNN+ BUCK + SAFF + SUNF	6	2
36	WG + WL + WB	All 9 warm-season species	9	3
37	WG + WL + WB	PROSO + CHICK + BUCK	3	3
38		SORG + COW + SAFF	3	3
39		TEFF + SUNN + SUNF	3	3
40	CG + CL + CB + WG + WL + WB	All 18 species	18	6

#### Seeding rates

Seeding rates for the different cover crops in pure stands were based on recommended broadcast rates [22] (Table 4). Cover crop mixture seeding rates were proportional to the rates used in pure stands. For example, in a three species mix, each species was seeded at one-third the full rate. The seeding rates for brassica species were reduced in the second year of this study, as the original seeding rate was greater than necessary to achieve maximum biomass.

**Table 4:** Seeding rates used for each cover crop species in pure stands.

Functional group	Species	Seeding rate (g∙m^-2^) *
CS-G	Barley	16.8
	Oats	16.8
	Wheat	16.8
CS-L	Austrian winter peas	11.2
	Red clover	1.7
	Yellow sweetclover	1.7
CS-B	Radish	1.7*
	Rapeseed	1.7*
	Turnip	1.7*
WS-G	Proso millet	2.8
	Sorghum sudangrass	5.6
	Teff	0.6
W-SL	Chickpea	16.8
	Cowpea	11.2
	Sunn hemp	5.6
WS-B	Buckwheat	11.2
	Safflower	2.8
	Sunflower	0.6

*Seeding rate decreased to 1.1 g∙m^-2^ in 2014.

#### Treatment establishment

Treatments were arranged in a randomized complete block design with four replications at each site with the exception of site 11, which had only three replications owing to space constraints. Plots were 5 x 10 m—though these dimensions varied slightly to accommodate corn and soybean row spacing at sites 4, 5, 6, 7, and 9. Seeds for each treatment were hand broadcast into a variety of field conditions—after small grains harvest, after corn harvest, and into maturing corn and soybeans. In some instances, harvested small grain fields were disked prior to cover crop seeding and establishment. In other instances, cover crop seeds were broadcast into standing stubble (Table 1). Field management decisions were left up to each cooperating farmer.

### Data collection

Cover crop aboveground biomass was harvested prior to winterkill. Where sufficient growth was present (sites 3, 10, and 11), weed aboveground biomass was also sampled. Biomass was sampled using two randomly placed 0.18 m^2^ quadrats in each plot for site 3 and one randomly placed 0.18 m^2^ quadrat in each plot for the rest of the sites harvested. For perspective, many plant diversity studies use a sample of 0.20 m^2^ per plot [23]. Cover crop biomass was separated to individual species. Weed biomass was also separated to individual species, with the exception of *Amaranthus spp*. and *Setaria spp*., which were separated to genus. Plant samples were oven-dried at 55°C for 7 days and dry mass determined.

### Data analysis

#### Diversity-productivity hypothesis

The diversity-productivity hypothesis was tested by calculating estimates of the effect size of increasing species and functional richness on biomass productivity. To separate the effects of species richness from the effects of functional richness, we asked the question: “Does increasing species richness without increasing functional richness increase aboveground biomass?” We approached this question in two ways: (1) by tripling the species richness within each functional group, and (2) by tripling the species richness of already functionally diverse mixtures. In the first case, for example, the difference between the biomass of the three-species grass mixture (treatment 11) and the average biomass of the constituent grasses grown in pure stands (treatments 2, 3, and 4—barley, oats, and wheat, respectively) was divided by the latter. This was also done for the three-species legume and brassica mixtures (treatment 12 and 13, respectively).

$$\mathrm{Effect}\;\mathrm{size}\;(\%)=\frac{B_{3\;\mathrm{species}\;\mathrm{mix}}‑{\bar{B}}_{\mathrm{pure}\;\mathrm{stand}}}{{\bar{B}}_{\mathrm{pure}\;\mathrm{stand}}}*\;100$$

In the second case, we compared the average aboveground biomass of treatments containing one cool-season grass, legume, and brassica (${\bar{B}}_{18,19,20}$) with treatment 17, which contained three cool-season grasses, three cool-season legumes, and three brassicas (*B*_*17*_).

$$\mathrm{Effect}\;\mathrm{size}\;(\%)=\frac{B_{17}‑{\bar{B}}_{18,19,20}}{{\bar{B}}_{18,19,20}}*\;100$$

To determine the effect of increasing functional richness, we held species richness constant and increased functional richness from one functional group to three. That is, we compared the aboveground biomass of treatments 11, 12, and 13 to treatments 18, 19, and 20.

$$\mathrm{Effect}\;\mathrm{size}\;(\%)=\frac{{\bar{B}}_{18,19,20}‑{\bar{B}}_{11,12,13}}{{\bar{B}}_{11,12,13}}*\;100$$

The effect of increasing species richness and functional richness was tested simultaneously by taking the aboveground biomass of the nine-species mixture (i.e., treatment 17) and subtracting the average aboveground biomass of those nine species in pure stands (i.e., treatments 2–10), and then dividing by the average aboveground biomass of the pure stands.

$$\mathrm{Effect}\;\mathrm{size}\;(\%)=\frac{B_{17}‑{\bar{B}}_{2‑10}}{{\bar{B}}_{2‑10}}*\;100$$

Calculating these values for each block at each site results in multiple estimates of effect size. We then applied simple one-sample t-tests to determine the effects of (1) increasing species richness alone, (2) increasing functional richness alone, and (3) increasing species and functional richness together. Due to irregularities in the warm-season species data, which will be discussed in the results, as well as the low number of replicates of these treatments, these treatments were excluded from the analysis, though treatment summary data are provided.

#### Diversity-invasibility hypothesis

**Fig 1:**
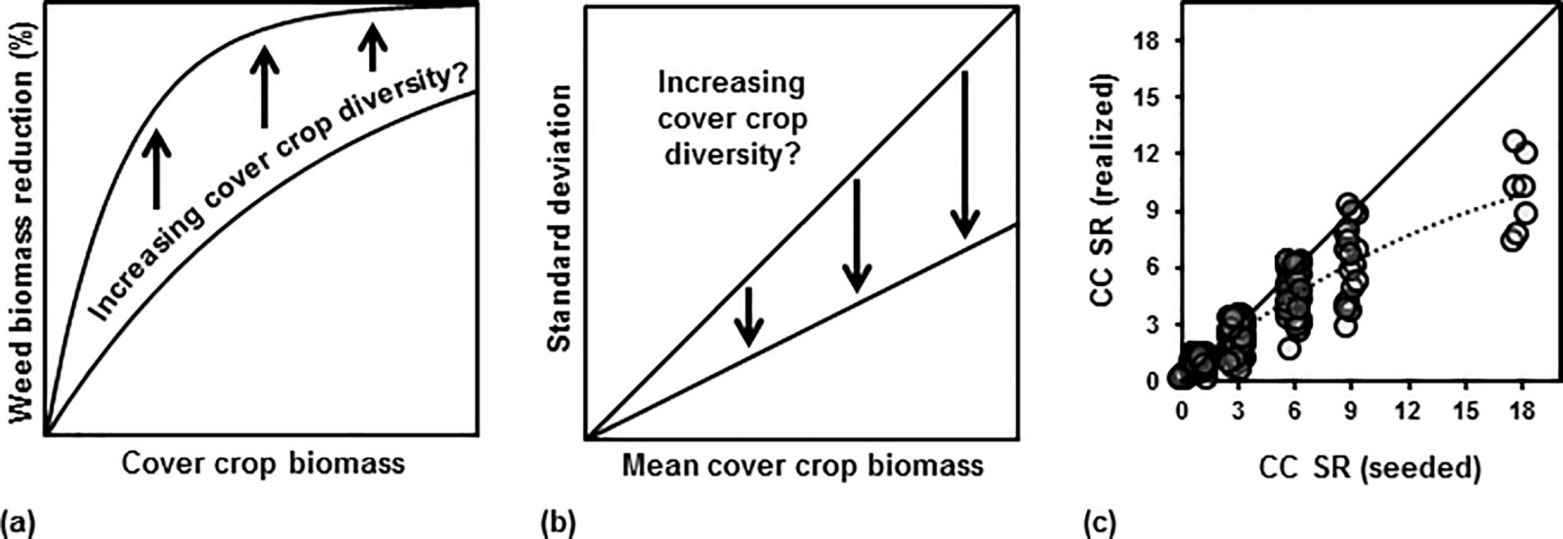
Hypothesized effect of species diversity on invasibility and stability. (a) Effect of increasing cover crop diversity (species or functional richness) on the relationship between weed biomass reduction and cover crop biomass. (b) Effect of increasing cover crop diversity on the relationship between standard deviation of cover crop biomass and mean cover crop biomass. (c) Realized cover crop species richness versus seeded cover crop species richness. Points jittered along both axes for ease of viewing. Solid line shows an idealized 1:1 relationship. Dashed line shows LOESS curve fitted to data (α = 1, λ = 2).

The diversity-invasibility hypothesis was tested by evaluating whether increasing cover crop diversity increased weed suppression of a cover crop on a per unit biomass basis (Fig 1A). To test this hypothesis, we first calculated percent weed biomass reduction (*BR*_*weed*_) as:

$${\mathrm{BR}}_{\mathrm{weed}}=\frac{{\bar{W}}_{\mathrm{control}}‑W}{W_{\mathrm{control}}}*\;100$$

Where ${\bar{w}}_{control}$ is the average weed biomass in the control (no cover crop) plots for each site and *w* is the weed biomass in each cover crop plot. Then, *BR*_*weed*_ was related to cover crop biomass (*x*) by an exponential equation:

$${\mathrm{BR}}_{\mathrm{weed}}=100‑100*e^{\beta_{1}x}$$

Where*𝛽*_*1*_ is a fitted parameter indicating the responsiveness of weed biomass to cover crop biomass—the larger the *𝛽*_*1*_ parameter, the more responsive weed biomass is to cover crop biomass. To assess whether species richness affects invasibility after controlling for the effect of cover crop biomass, a modified version of the equation was also fit:

$${\mathrm{BR}}_{\mathrm{weed}}=100–100\;*\;e^{\beta_{1}x+\beta_{2}xR}$$

Where *R* was either cover crop species richness or functional richness—as measured by the number of cover crop species or functional groups identified in the sampling quadrat—and *𝛽*_*2*_ was an additional fitted parameter that allowed for cover crop diversity to affect the relationship between percent weed biomass reduction and cover crop biomass. The significance of the parameter estimate *𝛽*_*2*_, based on an F-test, was used to draw conclusions about the impact of species richness and functional richness on invasibility.

#### Diversity-stability hypothesis

The term “stability” is used in the ecological and agricultural literature to refer to different ideas and is measured in different ways [24]. However, coefficient of variation (C_v_) of stand biomass, which is estimated as the sample standard deviation of the mean biomass (s) divided by the sample mean biomass ($\bar{x}$) is one of the most common ways to measure stability. A low C_v_ is considered an indicator of high stability and a high C_v_ an indicator of low stability. Generally, the C_v_ is then regressed on a diversity metric like species richness [25], with a negative slope indicating increased stability with increasing diversity. However, the results of this analysis can be misleading because the effects of diversity on stability can be confounded by a relationship between productivity and coefficient of variation [26]. To avoid this issue, our approach was to regress the standard deviation of cover crop biomass against mean cover crop biomass [27] for each treatment at each site and test whether increasing cover crop diversity—as measured by cover crop species and functional richness—decreased the slope of this relationship (Fig 1B). This assessed whether plot to plot variability within a field decreased with increasing cover crop diversity.

#### Seeded versus realized species richness

In analyzing the data, we had to decide whether to look at seeded diversity—how many species or functional groups were seeded—or realized diversity—how many species or functional groups were observed. Realized diversity typically correlates well to seeded diversity but the deviation between realized and seeded species richness tends to increase with increasing seeded species richness (Fig 1C).

When evaluating the effect of cover crop mixture diversity on weed suppression, we judged that realized diversity was the more appropriate metric to use—as any species or functional group that was seeded but absent in our sampling was unlikely to have an effect on the weed biomass in our sampling. However, using seeded diversity values instead of realized diversity values results in the same interpretive conclusions.

When evaluating the effect of diversity on stability, we judged that seeded diversity was the more appropriate metric to use—as the diversity-stability hypothesis is predicated on the idea that a more species-rich mixture is better insured against the failure of any one species. However, using realized diversity instead of seeded species richness also results in the same interpretive conclusions.

#### Statistical software

All statistical analyses were conducted using R 3.1.0 [28]. Non-linear regression models were fit with the nls2 package by Grothendieck [29]. Data and R code for the models fit can be found in the supplementary materials (S1 Dataset, S1 Key, S1 Code).

## Results

### Cover crop productivity by site

Cover crops were not harvested at 4 of the 11 sites seeded. At site 1, cover crop establishment was patchy throughout the site due to wheat stubble being swathed after cover crop seeding. At site 2, there was negligible cover crop growth due to extreme weed pressure. At sites 6 and 9 there was negligible cover crop growth (< 25 g m^-2^)—likely due to a combination of limited water and light under the standing corn crop and heat stress. Of those sites that were harvested, earlier seeding dates had the greatest aboveground biomass, with negligible biomass for those sites seeded after the beginning of September (S1 Fig).

### Cover crop productivity by treatment

Cover crop productivity by treatment varied widely across sites, but a few patterns were consistent across all sites: cool-season grasses and brassicas generally out-produced the cool-season legumes and warm-season grasses tended to out-produce the warm-season legumes (S2 and S3 Figs).

Cool-season mixtures tended to be dominated by brassicas and warm-season mixtures tended to be dominated by sorghum sudangrass and buckwheat, when present. A species’ biomass in pure stand was fairly predictive of its biomass in mixture, such that productive species in pure stands were also productive in mixture and vice versa.

### Effect of diversity on biomass productivity

**Fig 2:**
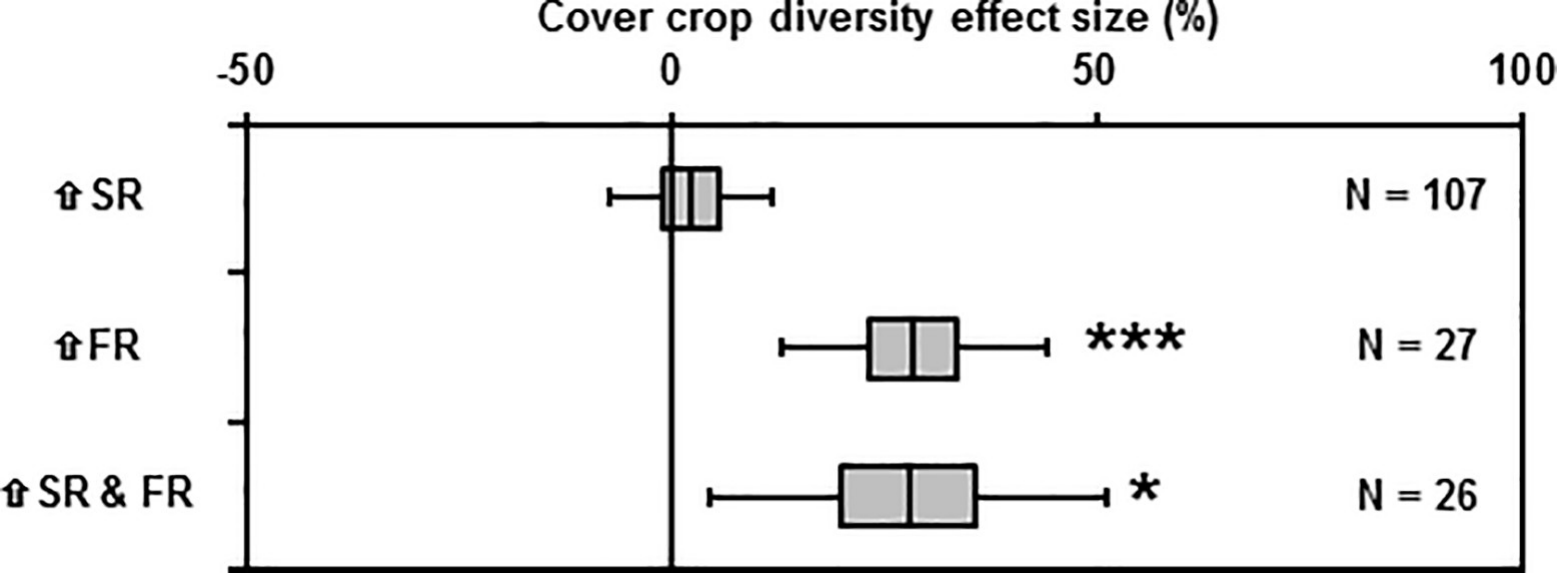
Effect of diversity on biomass productivity. Mean effect size of increasing cover crop diversity on cover crop productivity—specifically the effects of increasing species richness (⇧SR), increasing functional richness (⇧FR), and increasing both species and functional richness simultaneously (⇧SR & FR). Boxes and bars represent 50% and 95% confidence intervals, respectively. N = number of observations for each estimate. One observation is missing from the ⇧SR & FR category. Asterisks indicate *p*-value for the following test—H_0_: μ = 0; H_a_: μ ≠ 0. *P*-value > 0.05 (no asterisk); < 0.05(*); < 0.01(**); < 0.001(***).

Increasing species richness, while holding functional richness constant, did not increase average aboveground biomass (mean effect size = 2.3%, 95% C.I. = [-7.2, 11.9%], N = 107, *p*-value = 0.65). However, increasing functional richness, while holding species richness constant, increased average aboveground biomass by 29%, and increasing both functional and species richness simultaneously increased average aboveground biomass by 28% (Fig 2). At no site did a mixture produce more aboveground biomass than the most productive pure stand.

### Effect of diversity on weed suppression

**Fig 3:**
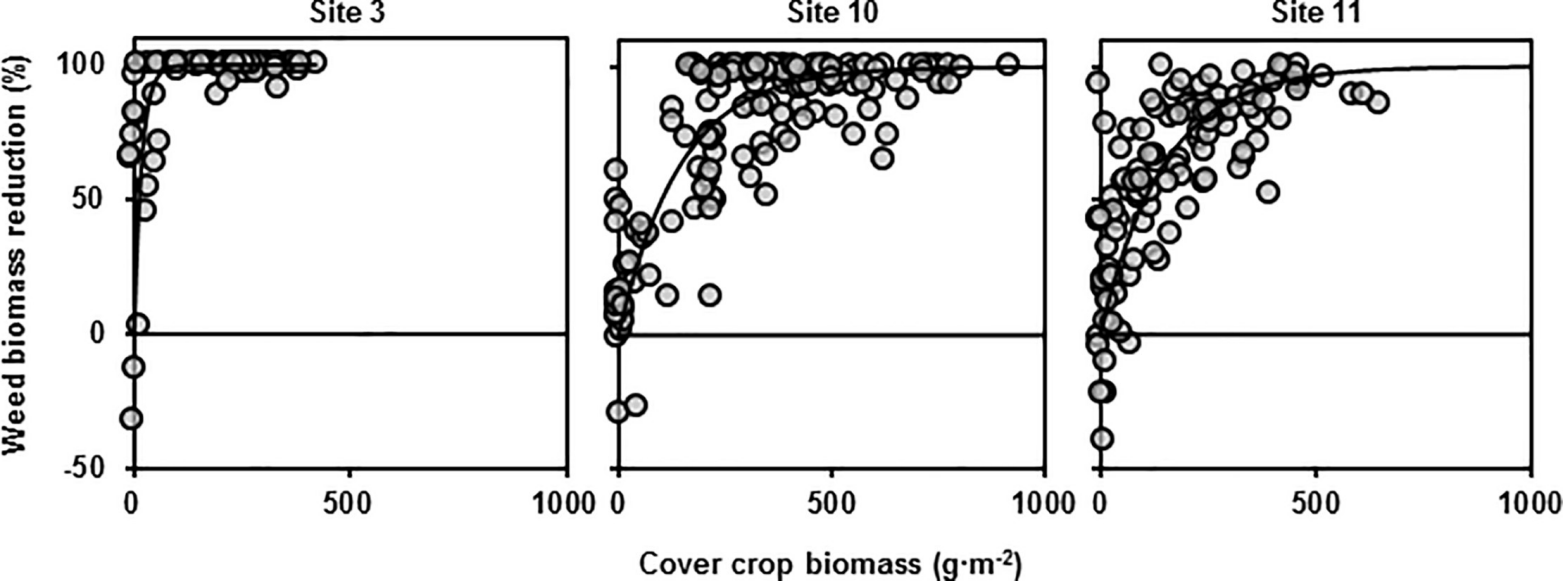
Effect of cover crop productivity on weed biomass reduction. Weed biomass reduction versus cover crop biomass at each of three sites. Exponential equation (Table 5) fit through each of the three data sets. Three data points with cover crop biomass beyond 1000 g m^-2^ not shown.

Increased cover crop biomass was associated with increased weed suppression at all three sites (Fig 3). However, neither adding cover crop species richness nor functional richness values improved the predictive results of the models tested, with the exception of adding functional richness to the site 10 base model, which resulted in a marginal improvement in predictive results (Table 5). Overall, the impression given is that increasing cover crop mixture diversity did not increase weed suppression.

**Table 5:** Parameter estimates for the exponential model fitted to weed biomass reduction versus cover crop biomass for each site with and without the inclusion of cover crop species richness (+SR) and functional richness (+FR) as a predictive variable along with F-test results. A significant value of ***β***_***2***_ shows that cover crop diversity affects the relationship between weed biomass reduction and cover crop biomass.

Site	Model	df	Parameter estimates±SEM * 10^3^^†^		RMSE	F-test results	
			*β*_*1*_	*β*_*2*_		F-value	*p*-value
3	Null	79	-57±12****	-	20.5	-	-
	+ SR	78	-30±18^NS^	-11±11^NS^	20.5	0.49	0.49
	+ FR	78	-74±37^NS^	16±33^NS^	20.5	<0.01	0.98
10	Null	159	-6.9±0.4****	-	17.1	-	-
	+ SR	158	-6.2±0.8****	-0.4±0.3^NS^	17.0	1.07	0.30
	+ FR	158	-4±1****	-1.9±0.8*	16.6	8.96	<0.01
11	Null	119	-6.8±0.5****	-	21.2	-	-
	+ SR	118	-7.5±0.9****	0.2±0.2^NS^	21.1	0.97	0.33
	+ FR	118	-7±1****	0.2±0.4^NS^	21.2	0.32	0.57

^†^Superscripts indicate *p*-values for the following hypothesis test—H_0_: parameter estimate = 0; H_a_: parameter estimate ≠ 0. *P*-value > 0.05(^NS^); < 0.05(*); < 0.01(**); < 0.001(***); < 0.0001(****).

### Effect of diversity on stability

**Fig 4:**
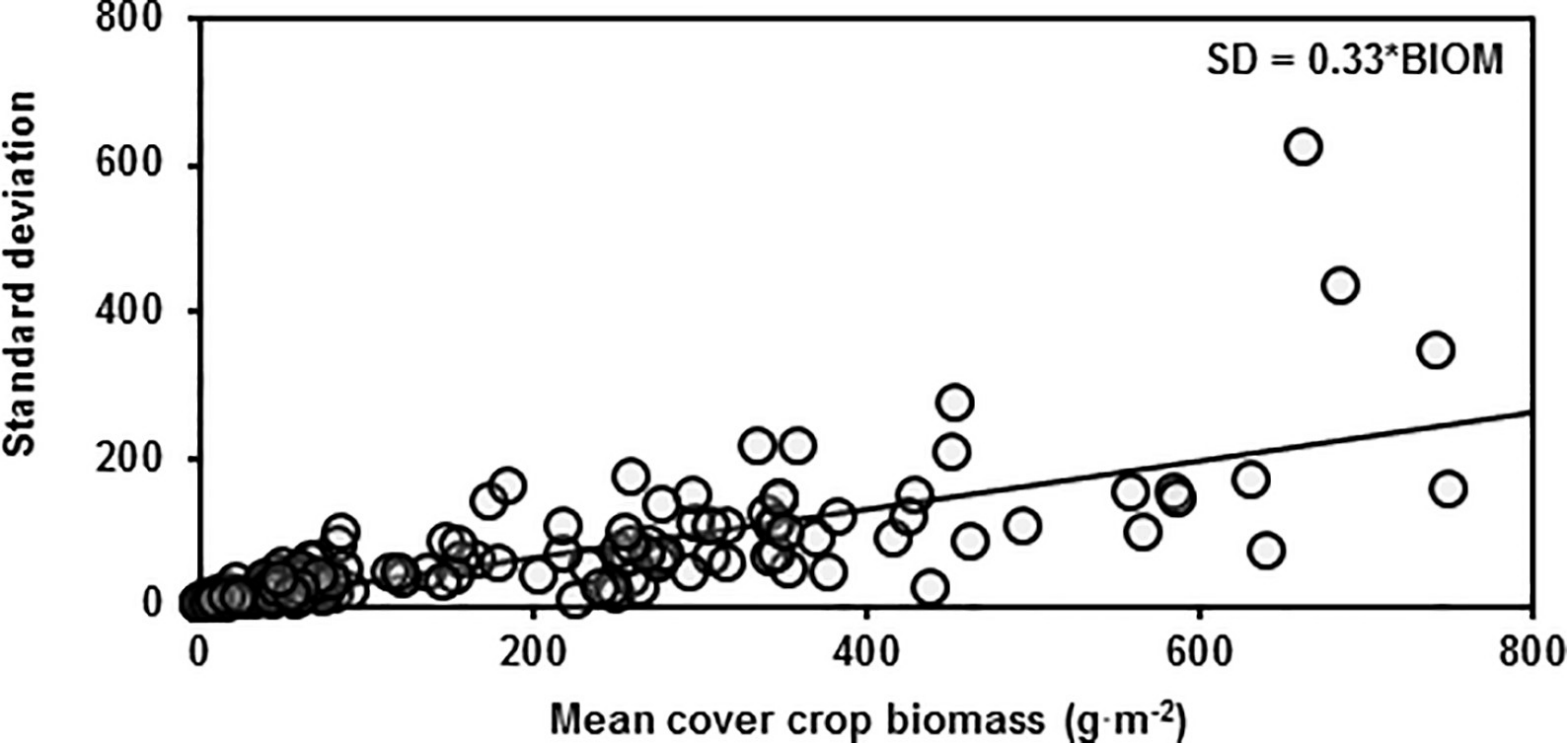
Stability of cover crop biomass. Standard deviation of cover crop aboveground biomass versus mean cover crop aboveground biomass for each treatment averaged across plots within each site. Line represents ordinary least squares regression with intercept term removed.

As mean cover crop biomass went up, so did the standard deviation (Fig 4). However, the slope of this relationship was not affected by cover crop mixture species richness or functional richness, suggesting that increasing cover crop mixture diversity does not stabilize biomass across individual sites (Table 6).

**Table 6:** Parameter estimates, degrees of freedom, and *p*-values for linear models relating standard deviation of cover crop biomass (SD) to mean cover crop aboveground biomass (BIOM) with and without cover crop species richness (SR) and functional richness (FR) interacting with cover crop aboveground biomass. A significant value of BIOM:SR or BIOM:FR shows that species or functional richness, respectively, affects the relationship between SD and mean cover crop biomass.

Equation^†^	df	Parameter^‡^	Estimate±SEM^§^	*p*-value
SD ~ BIOM (Base model)	172	BIOM	0.33**±**0.02****	<0.0001
SD ~ BIOM + BIOM:SR	171	BIOM	0.35**±**0.02****	<0.0001
		BIOM:SR	-0.006**±**0.005^NS^	0.23
SD ~ BIOM + BIOM:FR	171	BIOM	0.38**±**0.03****	<0.0001
		BIOM:FR	-0.03**±**0.01^NS^	0.07

^†^Standard deviations and mean biomass determined for each treatment across plots within each site.

^‡^Intercepts fixed to zero.

^§^Superscripts indicate *p*-values for the following hypothesis test—H_0_: slope = 0; H_a_: slope ≠ 0.

*P*-value > 0.05(^NS^); < 0.05(^*^); < 0.01(^**^); < 0.001(^***^); < 0.0001(^****^).

## Discussion

### Diversity-productivity hypothesis

The diversity-productivity hypothesis asserts that increased diversity should lead to increased average productivity, but it is predicated on the idea that diverse systems should have the potential to be more productive than even the most productive of pure stands by capturing a greater proportion of available resources via niche complementarity [30]. This disconnect between the theoretical underpinnings of the diversity-productivity hypothesis and the theoretical conclusions of the diversity-productivity hypothesis suggests that (1) we should be testing the theory of niche complementarity by testing whether increasing mixture diversity raises absolute productivity rather than average productivity and (2) niche complementarity is not the necessary conclusion to be drawn from the observation that increasing diversity increases average productivity.

Increasing cover crop mixture species and functional richness did not raise absolute productivity. The most productive single species at each site produced comparable amounts of biomass to the most productive mixture at each site. There was no evidence indicating that mixing species promoted niche complementarity and led to increased productivity of cover crop mixtures compared to single cover crop species.

Increasing cover crop mixture species richness and functional richness was associated with increased aboveground biomass. This meets the criteria of the diversity-productivity hypothesis, but we are reluctant to ascribe this effect to niche complementarity. Rather, the positive effect of increasing plant mixture diversity on average productivity can be explained by low biomass species pulling down the average biomass at low levels of diversity but not at high levels of diversity. The average productivity of pure stands and low functional richness category was brought down by the low biomass of legumes. In high diversity treatments, high biomass of grasses and brassicas compensated for the low biomass of the legumes. This is why mixing across functional groups led to increased average productivity but not mixing within a single functional group. Mixing grasses or brassicas with each other did not increase average productivity because there were no low biomass species being compensated for in the mixture. Similarly, mixing legumes together did not increase average productivity because there were no high producing species in the mixture to compensate for the low productivity of the legumes.

We would expect that had we increased seeding rates of the legumes beyond their recommended rate, we would have seen legume productivity increase and the strength of the relationship between diversity and average biomass productivity diminish. Indeed, He et al. [31] found that the positive relationship between diversity and productivity decreased with increasing plant density. This suggests that unproductive pure stands are not always at their maximum biomass when compared to mixtures.

Soil fertility likely played a role in the relative success of each of the cover crop species. There were no major soil nutrient deficiencies at any of the sites (data not shown). The most compelling agronomic results for the overyielding of mixtures is seen with grass-legume mixtures on soils that are not at optimum fertility. Fertilizing these soils leads to a reduced overyielding effect [32]. This is better evidence for niche complementarity. In this scenario, presence of the legumes, which are able to take advantage of atmospheric nitrogen, allow mixtures containing them to more fully exploit available nitrogen resources.

### Diversity-invasibility hypothesis

**Fig 5:**
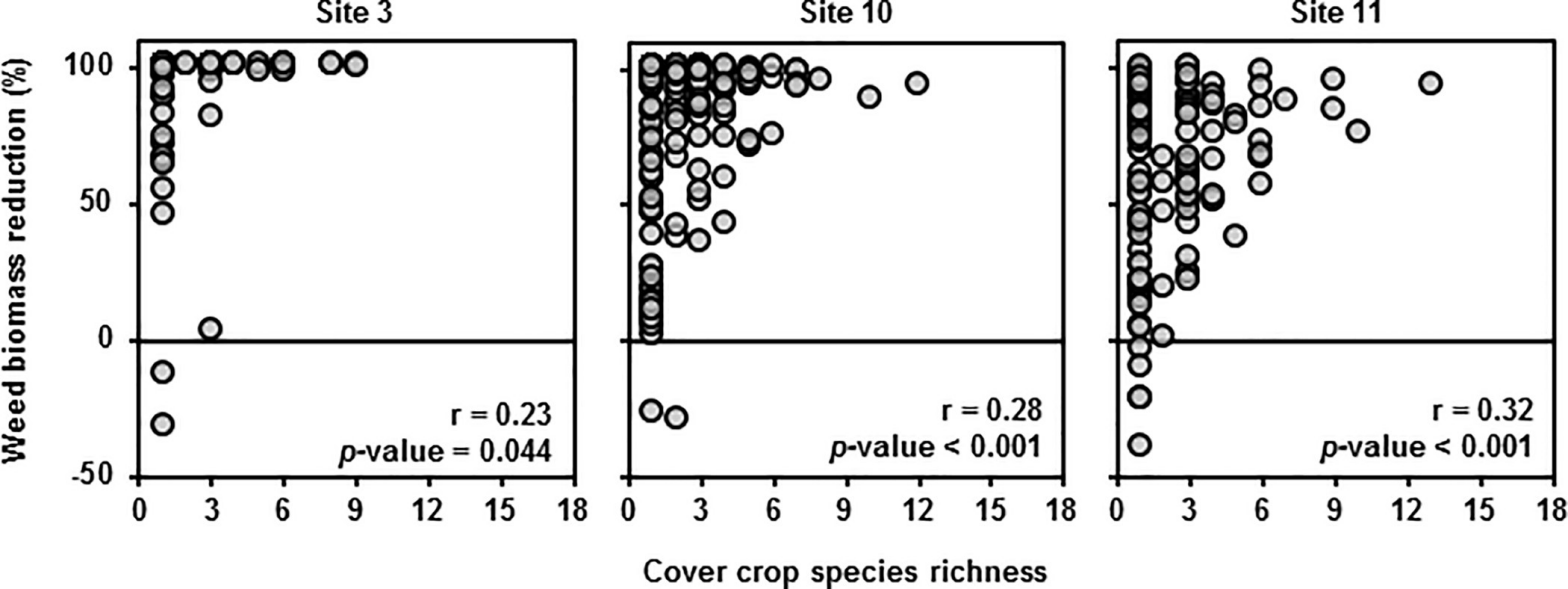
Effect of species diversity on weed biomass reduction. Weed biomass reduction versus realized cover crop species richness with Pearson correlation coefficients (r) for each site. *P*-values are for the following hypothesis test regarding the correlation coefficients—H_**0**_: r = 0; H_**a**_: r ≠ 0.

**Fig 6:**
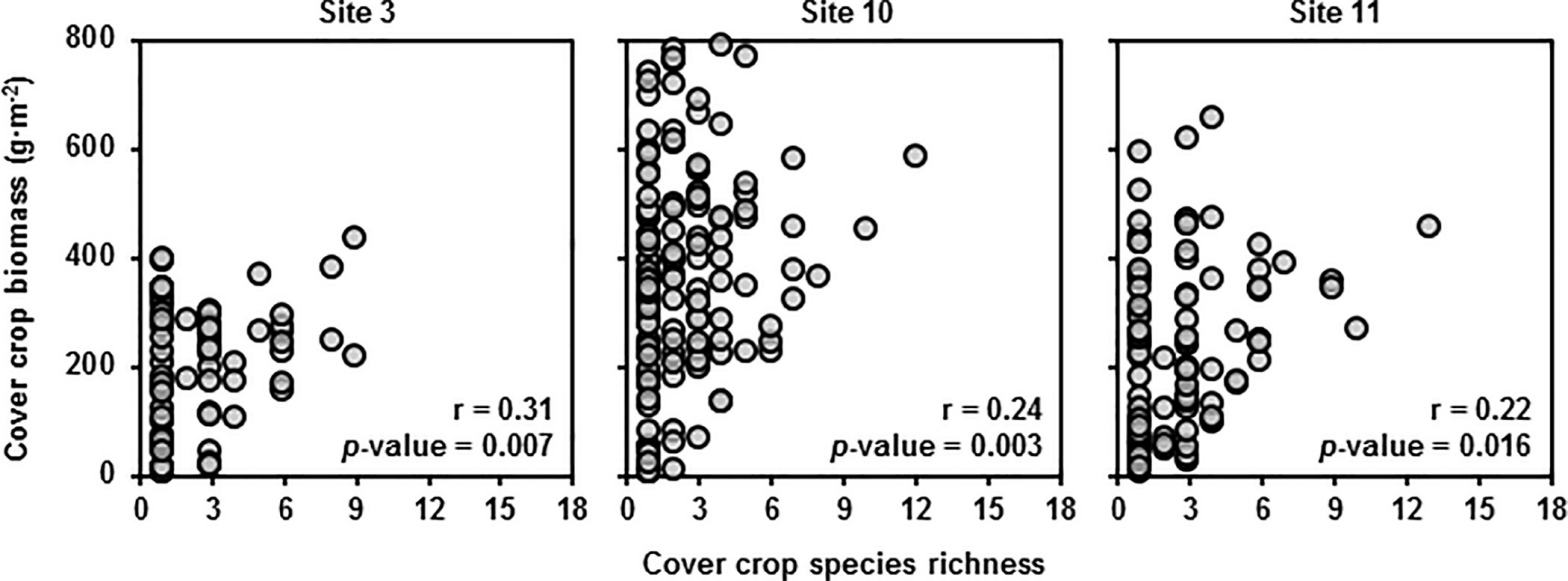
Cover crop biomass on species richness. Cover crop biomass versus realized cover crop species richness with Pearson correlation coefficients (r) for each site. Three data points with cover crop biomass beyond 1000 g m^**-2**^ not shown. *P*-values are for the following hypothesis test regarding the correlation coefficients—H_**0**_: r = 0; H_**a**_: r ≠ 0.

A common approach to evaluating the diversity-invasibility relationship is to evaluate an invasion resistance metric—e.g., weed biomass reduction—as a function of a diversity metric—e.g., cover crop species richness [16, 33–37]. This can gloss over the differences between correlation and causation, and can confound the effect of diversity with the effect of biomass productivity. If we analyze our data this way, we see that weed suppression is positively correlated with cover crop species richness (Fig 5). However, since cover crop aboveground biomass is also correlated with species richness (Fig 6), it is possible that the correlation between weed suppression and species richness is due to cover crop biomass rather than species richness. To determine whether species richness had an effect on weed suppression beyond its relationship with cover crop biomass, we first controlled the positive effect of cover crop productivity on weed suppression [38–39]. Controlling for that positive effect, we found that the effect of cover crop mixture diversity on weed suppression disappeared entirely in most cases.

In most manipulated plant diversity studies, as plant diversity increases so does average biomass productivity [23, 30]. Increased plant productivity is well documented to be associated with increased invasion resistance in native systems and increased weed suppression in agricultural systems [40–43]. Yet many diversity-invasibility studies gloss over the mediating effects of biomass productivity on invader suppression when discussing the correlation between plant mixture diversity and invasion resistance as evidence for the diversity-invasibility hypothesis. Subsequent meta-analyses that consolidate the findings of these studies also gloss over the confounding effects of biomass productivity on invader suppression [44, 45]. In the few studies where productivity is accounted for, the apparent effect of diversity on invasibility disappears [34, 46].

Reviews of mixed cropping literature often give the impression that it’s the actual mixing of crops that is promoting weed suppression [47–49] and rarely discuss how the increased average biomass of mixtures results in greater weed suppression. Furthermore, if we use the increased weed suppression of intercrops as evidence of increased resource use efficiency, that does not explain cases where sole crops are more suppressive than the intercrops [43, 50]. A better explanation is to look at variations in biomass where we observe that sole crops that are more weed suppressive than intercrops tend also to be more productive in terms of biomass.

Our study highlights an issue regarding the testing of the diversity-invasibility hypothesis—the covariance of diversity with productivity. Goldberg and Werner [51] made an early call for scientists to account for the effect of biomass when studying plant invasion, but it seems their advice has been largely ignored. After accounting for the effect of plant productivity on weed suppression in this study, we observed little effect of cover crop diversity on invasibility.

### Diversity-stability hypothesis

**Fig 7:**
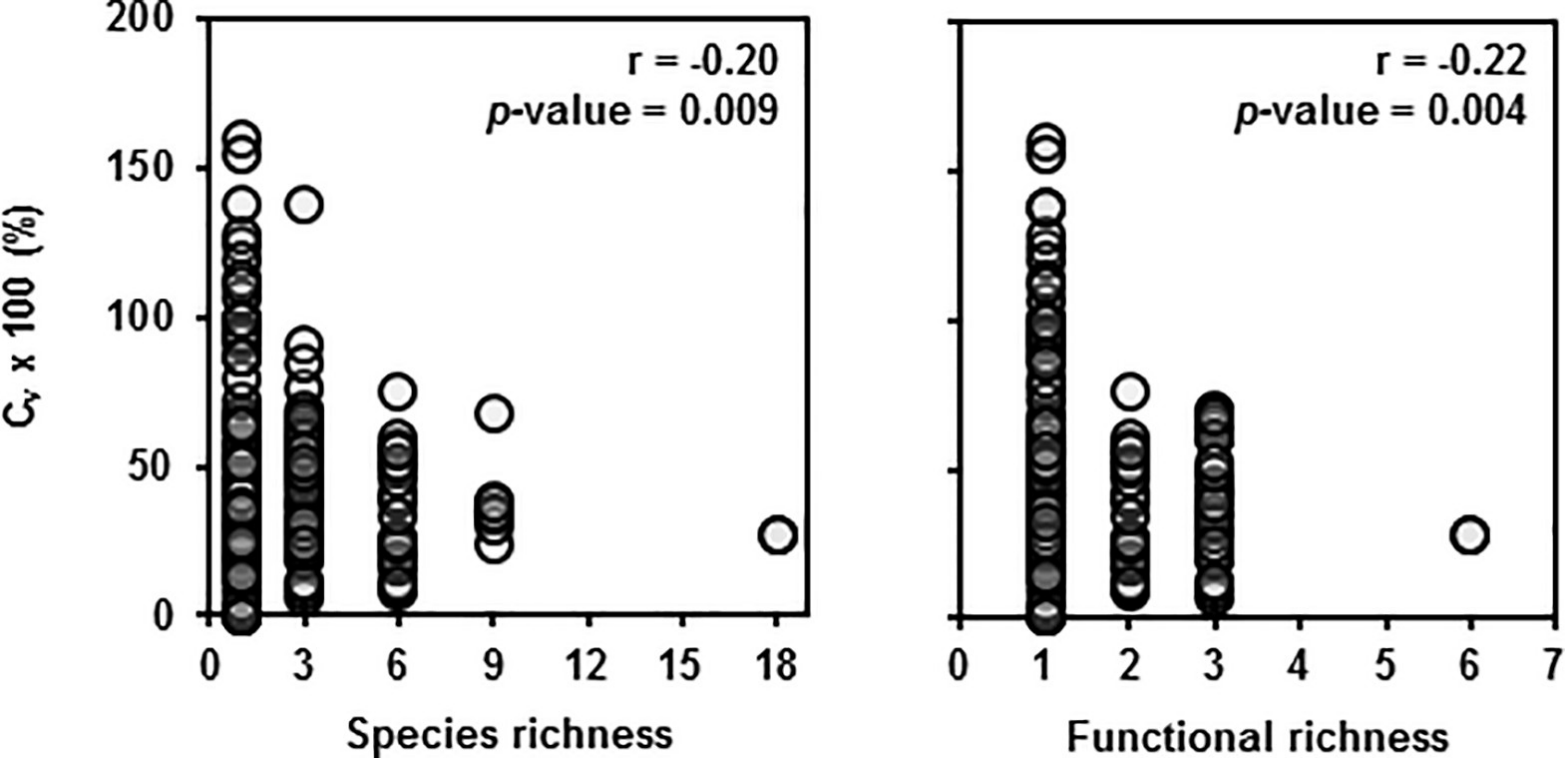
Effect of diversity measures on cover crop biomass coefficient of variation. Coefficient of variation of aboveground cover crop biomass across treatments and study sites plotted against realized species (left) and functional (right) richness. Pearson correlation coefficients (r) given with *p*-values for the following test—H_**0**_: r = 0; H_**a**_: r ≠ 0.

**Fig 8:**
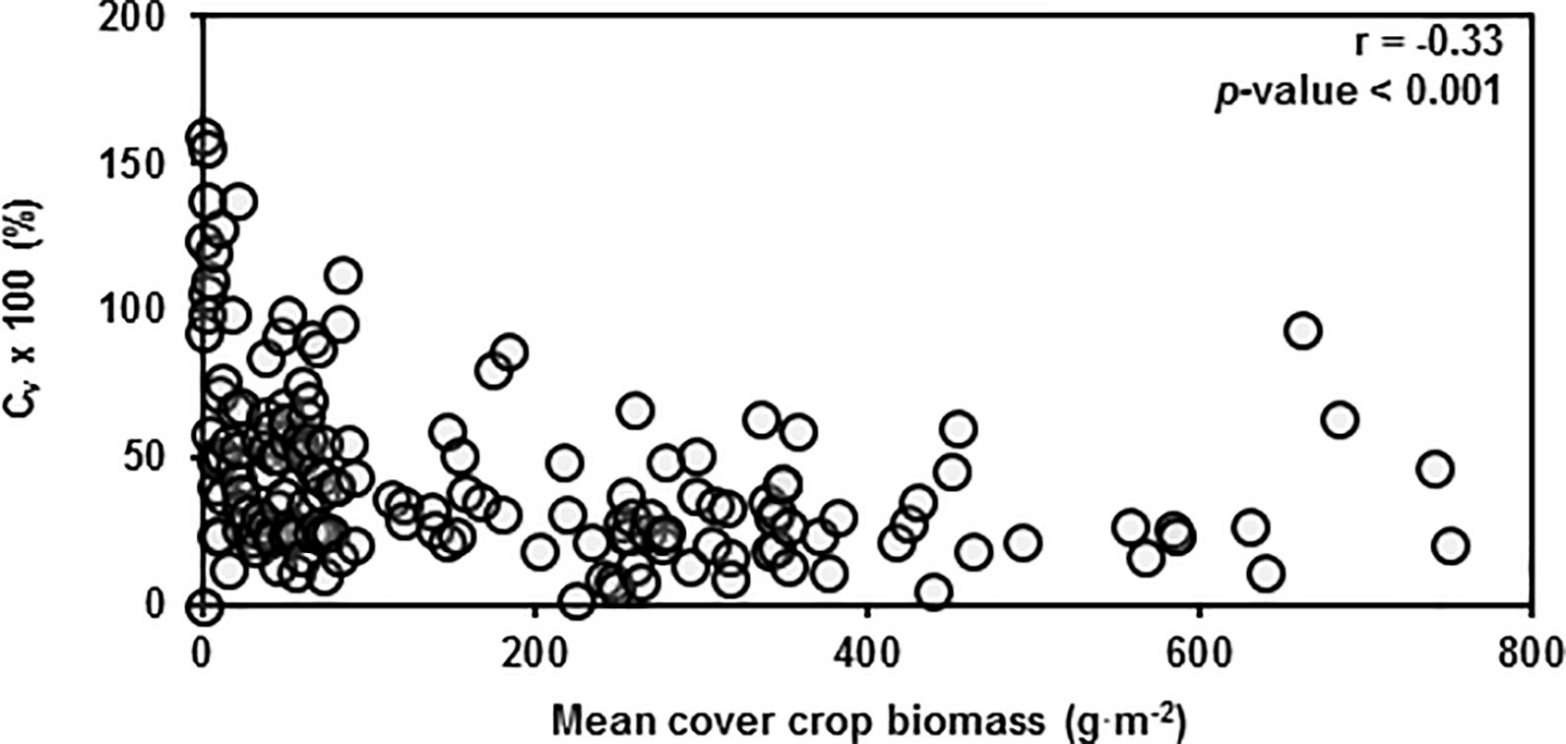
Cover crop coefficient of variation in relation to cover crop biomass. Coefficient of variation of aboveground cover crop biomass across treatments and study sites plotted against mean cover crop biomass. Pearson correlation coefficients (r) also given with *p*-values for the following test—H_**0**_: r = 0; H_**a**_: r ≠ 0.

Increasing diversity was correlated with decreases in Ĉ_v_ (Fig 7). However, this correlation was mediated through the negative relationship between Ĉ_v_ and mean cover crop biomass (Fig 8). If we look at the relationship between Ĉ_v_ and mean cover crop biomass, we find that at low biomass, the Ĉ_v_ tends to be greater and less consistent than at larger biomass. These results occur because small amounts of experimental error at high levels of mean biomass have marginal effects on Ĉ_v_, whereas at low levels of mean biomass, small amounts of error amplify into dramatic effects on Ĉ_v_. Thus, the correlation that exists between diversity and Ĉ_v_ is because low diversity treatments tended to have less biomass in our study and treatments with less biomass tended to have higher Ĉ_v_.

Studies have concluded that intercrops are more stable than sole crops on the basis of their C_v_ values being lower than those of the tested sole crops [32, 52]. However, in these studies intercrops were also more productive than sole crops. In cover crop mixture studies where the most diverse mixture was not the most productive treatment, neither were they the most stable [14, 17].

We found little evidence that increasing cover crop mixture diversity increased field-scale biomass stability. If we had greater species differentiation between the 18 species, as well as greater environmental heterogeneity, we might have expected a greater impact of diversity on stability. However, for the practical purposes of cover crop management, where our environmental conditions are relatively predictable and our suite of potential cover crops thrive and fail under relatively similar conditions, that point may be moot.

## Conclusions

While increasing cover crop mixture diversity was associated with increased average cover crop biomass productivity, we contest the traditional interpretation of this result as evidence of increased niche complementarity or resource use efficiency of diverse mixtures. We argue that increased niche complementarity or resource use efficiency of mixtures should be demonstrated by increased absolute productivity rather than average productivity, which we did not observe. Our results can be explained by the fact that the average biomass of pure stands was drawn down by low biomass species that were compensated for in mixture by high biomass species. While cover crop mixture diversity was positively related to metrics of invasion resistance and stability, we found these correlations to be driven by variation in cover crop biomass. Once we controlled for the effect of cover crop biomass, we found little evidence that cover crop mixture diversity positively affects invasion resistance or biomass stability. Mixing cover crops did not benefit biomass production, weed suppression, or biomass stability compared to a productive single species.

## Supporting information

S1 Dataset Cover crop and weed biomass data. (CSV)

S1 Key Descriptions for variable names presented in S1 Dataset. (DOCX)

S1 Code R code for models presented in the article. Code uses S1 Dataset. Variable name descriptions in S1 Key.
(DOCX)

S1 Fig Cover crop productivity and time of establishment. Boxplots of cover crop aboveground biomass for treatments #2–20 by seeding date. Seeding dates are not temporally equidistant.
(TIF)

S2 Fig Cool season cover crop productivity. Species specific cover crop biomass (±SEM) for treatments 2–20. Vertical dotted line separates pure stands (left) from mixtures (right). One extreme outlier (1156 g·m^2^) for rapeseed was omitted from the bar chart for Site 11. BAR = barley. OAT = oat. WHT = wheat. PEA = Austrian winter pea. RED = Red clover. YEL = Yellow sweetclover. RAD = Radish. PARE = Rapeseed. TURN = turnip.
(TIF)

S3 Fig Warm season cover crop productivity. Species-specific cover crop biomass (±SEM) for treatments 21–39 by site. Deer grazed on the sunflower plants prior to sampling at site 11 but not site 3. Sampling at sites 3 and 11 happened after some of the warm-season species began to shed their foliage, leading to lower measured aboveground biomass than was actually produced. Vertical dotted line separates pure stands (left) from mixtures (right). PROSO = proso millet. SORG = Sorghum sudangrass. TEFF = teff. CHICK = chickpea. COW = cowpea. SUNN = sunn hemp. BUCK = buckwheat. SAFF = safflower. SUNF = sunflower.
(TIF)
