# Supplementary material for: Cover crop mixture diversity, biomass productivity, weed suppression, and stability
Source: PLoS One. 2019 Mar 14;14(3):e0206195. doi: 10.1371/journal.pone.0206195 (PMC6417710; doi:10.1371/journal.pone.0206195)
Supplement: S1 Key — (DOCX) [file pone.0206195.s002.docx]

**S1 Key.** Descriptions for variable names presented in S1 Dataset.

| **Variable name** | **Description** | |
| --- | --- | --- |
| obs.no | Observation number | |
| site.no | Site number | |
| site.name | Site name code | |
| plant.date | Cover crop planting date | |
| block | Block | |
| func.grps | Number of cover crop functional groups seeded and realized | |
| spp.no | Number of cover crop species seeded | |
| plant.samp.date | Aboveground biomass sampling date | |
| dm.bar.g | Species specific cover crop dry matter sampled (g) | Barley |
| dm.oat.g |  | Oats |
| dm.wht.g |  | Wheat |
| dm.pea.g |  | Austrian winter peas |
| dm.red.g |  | Red clover |
| dm.yel.g |  | Yellow sweetclover |
| dm.rad.g |  | Radish |
| dm.rape.g |  | Rapeseed |
| dm.turn.g |  | Turnip |
| dm.proso.g |  | Proso millet |
| dm.sorg.g |  | Sorghum sudangrass |
| dm.teff.g |  | Teff |
| dm.chick.g |  | Chickpea |
| dm.cow.g |  | Cowpea |
| dm.sunn.g |  | Sunn hemp |
| dm.buck.g |  | Buckwheat |
| dm.saff.g |  | Safflower |
| dm.sunf.g |  | Sunflower |
| dm.COV.g | Cover crop dry matter sampled (g) |  |
| dm.W.g | Weed dry matter sampled (g) |  |
| dm.TOT.g | Total dry matter sampled (g) |  |
| quad.size.m2 | Quadrat size (m^2^) | |
| quad.toss | Number of quadrat tosses | |
| samp.area.m2 | Sampling area (m^2^) = Quadrat size (m^2^) * Number of quadrat tosses | |
| dm.COV.g.m2 | Cover crop dry matter (g/m^2^) | |
| dm.W.g.m2 | Weed dry matter (g/m^2^) | |
| dm.TOT.g.m2 | Total dry matter (g/m^2^) | |
| W.YL | Weed yield loss (%) | |
| SR.cov | Cover crop species richness (realized) | |
