# Supplementary material for: Cover crop mixture diversity, biomass productivity, weed suppression, and stability
Source: PLoS One. 2019 Mar 14;14(3):e0206195. doi: 10.1371/journal.pone.0206195 (PMC6417710; doi:10.1371/journal.pone.0206195)
Supplement: S1 Code — Code uses S1 Dataset. Variable name descriptions in S1 Key. (DOCX) [file pone.0206195.s003.docx]

S1 Code. R code for models presented in the article. Code uses S1 Dataset. Variable name descriptions in S1 Key.

ccdiv<-read.csv("S1_Dataset.csv", header=TRUE)
ccdiv<-ccdiv[ccdiv$site.no == 3| ccdiv$site.no == 10| ccdiv$site.no == 11 ,]

library(nls2) # non-linear model fitting package

library(plyr) # contains rename function
library(raster) # contains cv function

# Seeded versus realized species richness #

plot(jitter(SR.cov, 2)~jitter(spp.no,2), data=ccdiv,
 xlab = "Number of cover crop species seeded",
 ylab = "Number of cover crop species present",
 xlim = c(0,19), ylim = c(0,19),
 xaxt = 'n', yaxt = 'n')
axis(side = 1, at = seq(0,18,3), labels = seq(0,18,3))
axis(side = 2, at = seq(0,18,3), labels = seq(0,18,3))
abline(a=0, b=1, lty="dashed")
loess.fit<-loess.smooth(ccdiv$spp.no, ccdiv$SR.cov, span = 1,
 degree=2, evaluation=100, family = "gaussian")
lines(loess.fit, col = "black")

# Diversity-Invasibility Hypothesis Test #

ccdiv <-read.csv("S1_Dataset.csv", header=TRUE)
ccdiv <-ccdiv[ccdiv$trt.no > 1,] # removes control plots
ccdiv <-ccdiv[ccdiv$site.no == 3,] # Use to select site (options=3,10,11)

# Simple model: Weed yield loss (YL) vs cover crop biomass (X) #
YL <- ccdiv$W.YL
X <- ccdiv$dm.COV.g.m2
mod.simp <-nls2(YL ~ 100-100*(exp(b*X)),
 start = list (b=0),
 trace = TRUE)

summary(mod.simp) # model parameters

SS.simp <- sum((predict(mod.simp) - YL)^2) # sum of squares
df.simp <- nrow(ccdiv)-1 # degrees of freedom
sqrt(mean((YL-predict(mod.simp))^2)) # RMSE

# Complex model: Adding in cover crop SR and FR #
YL <- ccdiv$W.YL
X <- ccdiv$dm.COV.g.m2
SR <- ccdiv$SR.cov
FR <- as.numeric(ccdiv$func.grps)
mod.comp <-nls2(YL ~ 100-100*(exp(b*X+c*X*SR)),
 start = list (b=0, c = 0),
 trace = TRUE) # Use to switch between SR and FR.

summary(mod.comp) # model parameters

SS.comp <- sum((predict(mod.comp) - YL)^2) # sum of squares
df.comp <-nrow(ccdiv)-2 # degrees of freedom
sqrt(mean((YL-predict(mod.comp))^2)) # RMSE

# F-test
df.num = df.simp - df.comp
df.den = df.comp
F <-((SS.simp-SS.comp)/SS.comp)/((df.simp-df.comp)/df.comp) # F-value
p <- 1-pf(q=F, df1=df.num, df2=df.den) # p-value

# Diversity-Stability Hypothesis Test #

ccdiv <- read.csv("S1_Dataset.csv", header=TRUE)
ccdiv <- ccdiv[complete.cases(ccdiv$dm.TOT.g.m2),]
ccdiv <- ccdiv[ccdiv$trt.no > 1,]

temp <- as.data.frame(aggregate(x = ccdiv$dm.COV.g.m2,
 by = list(ccdiv$trt.no,
 ccdiv$site.no,
 ccdiv$spp.no,
 ccdiv$func.grps),
 FUN = function(x)
 { c(BIOM=mean(x) , SD=sd(x))}))
temp <- rename(temp, c("Group.1" = "trt.no",
 "Group.2" = "site.no",
 "Group.3" = "SR",
 "Group.4" = "FR",
 "x" = "x"))
temp$BIOM <- temp$x[,1]
temp$SD <- temp$x[,2]

summary(lm(SD~0 + BIOM, data = temp))

summary(lm(SD~0 + BIOM + BIOM:SR, data = temp))

summary(lm(SD~0 + BIOM + BIOM:FR, data = temp))
