# Supplementary figures and images for: Cover crop mixture diversity, biomass productivity, weed suppression, and stability
Source: PLoS One. 2019 Mar 14;14(3):e0206195. doi: 10.1371/journal.pone.0206195 (PMC6417710; doi:10.1371/journal.pone.0206195)

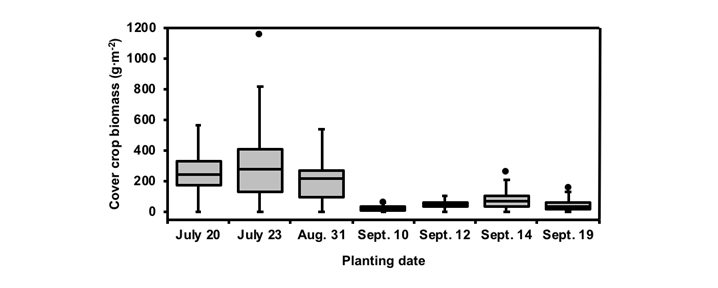

Supplement: S1 Fig — Boxplots of cover crop aboveground biomass for treatments #2–20 by seeding date. Seeding dates are not temporally equidistant. (TIF) [file pone.0206195.s004.tif]

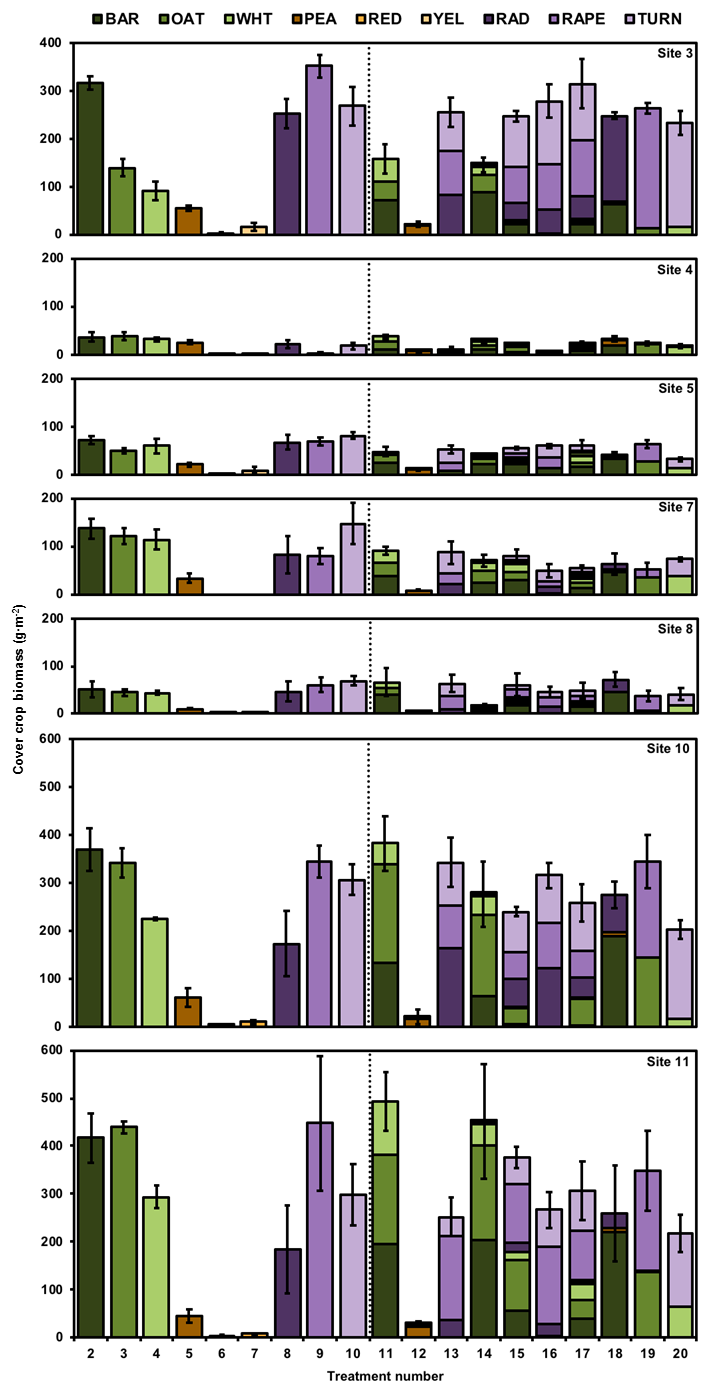

Supplement: S2 Fig — Species specific cover crop biomass (±SEM) for treatments 2–20. Vertical dotted line separates pure stands (left) from mixtures (right). One extreme outlier (1156 g·m2) for rapeseed was omitted from the bar chart for Site 11. BAR = barley. OAT = oat. WHT = wheat. PEA = Austrian winter pea. RED = Red clover. YEL = Yellow sweetclover. RAD = Radish. PARE = Rapeseed. TURN = turnip. (TIF) [file pone.0206195.s005.tif]

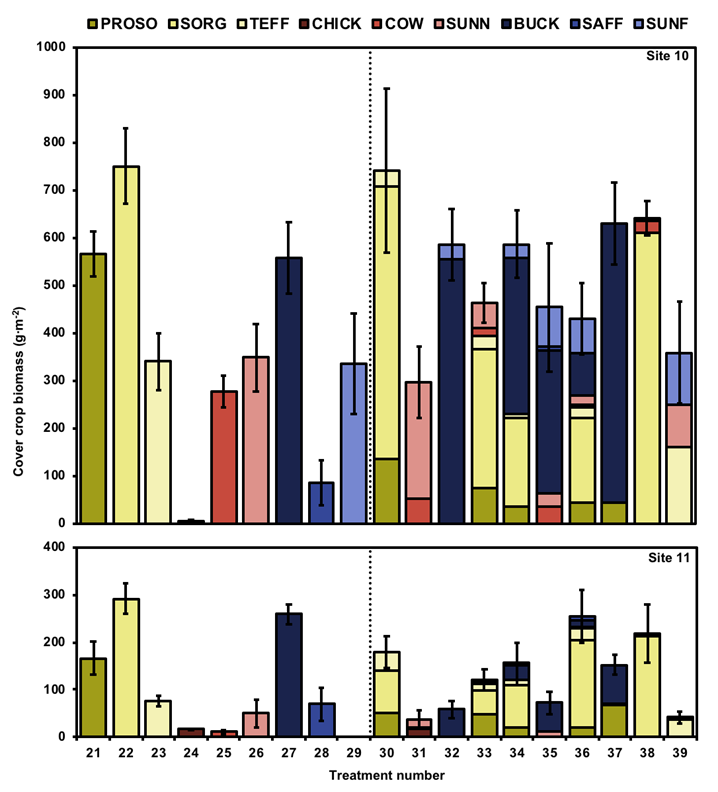

Supplement: S3 Fig — Species-specific cover crop biomass (±SEM) for treatments 21–39 by site. Deer grazed on the sunflower plants prior to sampling at site 11 but not site 3. Sampling at sites 3 and 11 happened after some of the warm-season species began to shed their foliage, leading to lower measured aboveground biomass than was actually produced. Vertical dotted line separates pure stands (left) from mixtures (right). PROSO = proso millet. SORG = Sorghum sudangrass. TEFF = teff. CHICK = chickpea. COW = cowpea. SUNN = sunn hemp. BUCK = buckwheat. SAFF = safflower. SUNF = sunflower. (TIF) [file pone.0206195.s006.tif]
